# Effectiveness of Adaptive Digital Interventions Triggered by Passive Sensing for Sleep Improvement in Adults: A Systematic Review and Meta-Analysis

**DOI:** 10.7759/cureus.105457

**Published:** 2026-03-18

**Authors:** Anees A Alyafei, Aysha MA Hussein, Sara Tariq Al Abdulla

**Affiliations:** 1 Wellness Programs, Preventive Medicine, Primary Health Care Corporation, Doha, QAT

**Keywords:** adaptive digital interventions, just-in-time adaptive interventions, passive sensing, personalized sleep therapy, sleep disorders, sleep hygiene interventions, sleep status, smart health wearable, wearable sleep technology

## Abstract

Adaptive digital interventions that respond to real-time physiological data from passive sensors are emerging as personalized tools for sleep improvement. The aim of this systematic review and meta-analysis was to evaluate the effectiveness of these interventions in improving sleep outcomes and overall health indicators in adults. A comprehensive literature search was conducted across PubMed, Embase, Cochrane CENTRAL, and ScienceDirect for studies published from January 2015 to July 2025. The included studies were randomized controlled trials (RCTs) involving adults (≥18 years), with or without diagnosed sleep disorders, evaluating adaptive digital interventions triggered by passive sensing technologies (actigraphy, wearables, smartphones), compared to static digital tools, usual care, or waitlist controls. The outcomes had to include at least one sleep-related or secondary health metric. Two reviewers independently screened the studies, extracted data, and assessed the risk of bias using the Cochrane RoB 2 tool. Meta-analyses were conducted using random effects models. Effect sizes were expressed as standardized mean differences (SMDs) with 95% confidence intervals (CIs). Heterogeneity was evaluated using the I² statistic. Twelve RCTs (n = 798 participants) were included. Adaptive interventions significantly improved wakefulness after sleep onset (WASO, SMD = 3.22; 95% CI: 3.02-3.41), with moderate heterogeneity (I² = 70.7%). The effects on the Pittsburgh Sleep Quality Index (PSQI) score, sleep efficiency, and latency were small and not significant. However, the secondary outcomes, including improvements in quality of life (SMD = 1.36), depressive symptoms (SMD = 0.53), sleep duration (SMD = 0.41), and neuropsychiatric inventory scores (SMD = -1.21), were favorable. Subgroup analyses revealed greater benefits in populations with cognitive impairments and interventions using advanced sensing tools (MotionWatch8). Adaptive digital interventions triggered by passive sensing show promise for reducing night-time awakenings and enhancing mood and quality of life. Their utility may be greatest in cognitively vulnerable populations. Further research is needed to optimize adaptivity algorithms, ensure sustained engagement, and assess long-term outcomes in real-world settings.

## Introduction and background

Sleep disorders are a burgeoning public health concern, with nearly one-third of the global adult population being affected. Insomnia, obstructive sleep apnea, delayed sleep phase syndrome, and general sleep disturbances are associated with a broad array of adverse outcomes, including impaired cognitive function, an increased risk of cardiovascular and metabolic disorders, mood disturbances, and a compromised quality of life [1]. The World Health Organization and other global health bodies have recognized poor sleep as a modifiable risk factor for principal noncommunicable diseases, which highlights the importance of the discovery of effective, scalable, and accessible interventions. Poor sleep affects a substantial proportion of adults worldwide, with epidemiological surveys suggesting that up to one-third of the global population experiences chronic sleep disturbance or clinically significant insomnia symptoms. These disturbances are not benign; they are associated with increased risks of cardiovascular disease, type 2 diabetes, and obesity, as well as higher rates of depression, anxiety, cognitive decline, and reduced quality of life [2].

Over the past decade, digital health interventions have been increasingly promoted as hopeful solutions to enhance sleep problems. Traditional digital interventions, such as internet-delivered cognitive behavioral therapy for insomnia (CBT-I), smartphone-based mindfulness training, and sleep hygiene programs of fixed duration, hold advantages in terms of scalability, cost, and user convenience [3]. However, while they have been proven effective, such fixed digital programs often exhibit suboptimal levels of engagement and adherence, partly due to their failure to account for the dynamic and context-dependent nature of people's sleep behavior and patterns [4].

In response to these limitations, there is growing interest in adaptive digital interventions (ADIs), programs that modify their therapeutic content or delivery in real time based on user data. ADIs leverage passively collected signals - such as movement patterns from actigraphy or wearables, heart rate and heart rate variability, smartphone usage, and ambient light exposure - to infer an individual's current sleep-wake state, circadian rhythm, and level of arousal. These real-time data streams are then used to trigger just-in-time prompts or to adjust the timing, intensity, or content of therapeutic modules (e.g., delivering relaxation exercises after a fragmented night or advancing light exposure when a delayed sleep phase is detected), thereby personalizing support to the user's momentary context [5,6].

When combined with passive sensing technologies, such as actigraphy, smartphone sensors, or wearables, these interventions can passively monitor users' behaviors and physiological states (e.g., movement, heart rate variability, sleep latency) in real time without the need for active input [7]. This enables interventions to be personalized based on contextual factors such as sleep regularity, circadian rhythm, or sleep environment disruption. Through the reduction of user burden and maximization of ecological validity, passive sensing lends itself to the concept of just-in-time adaptive interventions (JITAI) [8,9]. These systems can deliver sleep prompts, recommendations, or behavioral nudges at the most relevant time, for example, cuing presleep wind-down activities during periods of increased physiological arousal or scheduling digital CBT-I modules following a bad night's sleep [10]. The use of machine learning models for real-time analysis also allows these tools to learn and optimize delivery approaches in an ongoing manner, with considerable potential for improving both short-term sleep outcomes and long-term behavior change [11].

While adaptive digital sleep interventions have been created and distributed quickly, their efficacy has been scientifically assessed in a piecemeal fashion [12]. While many individual studies and pilot trials have shown that subjective sleep quality and sleep-related outcomes (e.g., sleep efficiency, sleep onset latency, and daytime fatigue) improved, the findings have been heterogeneous, and the methodological rigor has differed considerably. Moreover, existing meta-analyses of e-sleep treatments have largely examined static programs (e.g., CBT-I web modules or apps) and have not systematically investigated the added value of adaptivity or the use of passive sensing technologies as triggers for personalization.

There are also important questions about which populations benefit most from these adaptive strategies (e.g., adults with insomnia as a clinical problem vs. more general sleep disturbance or younger technology-savvy groups vs. older adults). Finally, the degree to which different types of sensing inputs (e.g., actigraphy vs. smartphone-based sensors) influence intervention efficacy has not been widely compared.

As the number of digital interventions claiming to use artificial intelligence, biofeedback, or real-time sensing to tailor interventions grows, a unified, methodologically strict review and meta-analysis is critically necessary. Such a synthesis could help inform clinical practice, regulatory policy, and future digital health innovation by clarifying what works best, under what conditions, and for whom. Previous systematic reviews of digital sleep interventions have largely focused on static programs, such as standard web-based or app-delivered CBT-I, in which content is pre-scheduled and does not adapt to real-time user data. In contrast, the present review specifically examines ADIs that are dynamically informed by passive sensing, aiming to clarify whether this additional layer of personalization confers measurable benefits for sleep and related health outcomes.

The primary objective is to evaluate how effective adaptive digital sleep interventions, activated by passive sensing technologies, are at improving sleep-related outcomes in adults. In particular, the review combines the following quantitative evidence:

Changes in subjective and objective measures of sleep quality (e.g., the Pittsburgh Sleep Quality Index (PSQI) and actigraphy-measured sleep efficiency), modifications to some sleep variables, such as sleep onset latency, wake after sleep onset (WASO), and total sleep time, and secondary effects include daytime fatigue, mood, adherence to intervention, and user satisfaction.

A secondary objective is to examine the heterogeneity of effects across subgroups, such as by type of sleep disorder (e.g., insomnia vs. general disturbances), intervention modality (e.g., CBT-I vs. mindfulness), and sensing method (e.g., wearables vs. smartphones). This review also extracts standard design features of effective interventions and describes gaps in current research to guide the future development of adaptive digital therapeutics for sleep.

## Review

Methodology

The meta-analysis and systematic review were carried out following the Preferred Reporting Items for Systematic Reviews and Meta-Analyses (PRISMA) 2020 statement [13]. The protocol was prospectively registered in the International Prospective Register of Systematic Reviews (PROSPERO) under ID CRD420251137434. The PRISMA checklist is provided in Appendix 3. PICO format (Population, Intervention, Comparison, Outcome) organized the research question and the analysis (Table 1). 

**Table 1:** The PICO framework for the effectiveness of adaptive digital interventions triggered by passive sensing for sleep improvement in adults PICO: Population, Intervention, Comparison, Outcome; CBT: Cognitive Behavioral Therapy

PICO Element	Description
Population	Adults aged ≥18 years, with or without diagnosed sleep disorders (e.g., insomnia, circadian rhythm disorders, general sleep disturbance), including cognitively vulnerable populations such as mild cognitive impairment, dementia, depression, and cancer-related sleep problems.
Intervention	Adaptive digital interventions triggered by passive sensing technologies (e.g., wearables, actigraphy, smartphone or ambient sensors) that dynamically tailor content or timing of sleep-related interventions (e.g., CBT-I modules, sleep hygiene education, relaxation cues, light therapy) based on real-time physiological or behavioral data (just-in-time adaptive interventions).
Comparator	Static (non-adaptive) digital interventions, usual care, wait-list controls, sham or attention controls, or non-digital behavioral interventions without real-time personalization or sensor-triggered adaptation.
Outcomes	Primary outcomes: Sleep quality using the Pittsburgh Sleep Quality Index​​​, sleep efficiency, sleep latency, wake after sleep onset. Secondary outcomes: Total sleep time, sleep duration, daytime fatigue, depressive symptoms, quality of life, neuropsychiatric outcomes, and intervention adherence/engagement metrics.

Eligibility Criteria

We included randomized controlled trials (RCTs) published between January 2015 and July 2025 that aimed to evaluate digital interventions for sleep improvement among adults (≥18 years). Eligible participants were from either the general population or included individuals who had been clinically diagnosed with sleep disorders, such as insomnia, obstructive sleep apnea, or circadian rhythm disorders. Interventions had to leverage passive sensing technologies, such as actigraphy, wearable sensors, smartphone apps, or ambient sensors, to deliver adaptive or semiadaptive content; this included real-time personalized features, such as CBT-I modules, sleep hygiene reminders, relaxation cues, or light exposure manipulation. Acceptable comparators included static digital interventions (nonpersonalized apps), usual care, waitlists, or nondigital behavioral therapies. Trials were required to have reported a minimum of one of the following outcomes: sleep quality (e.g., PSQI), sleep efficiency, sleep latency, WASO, daytime functioning, fatigue, depressive or anxiety symptoms, total wake time, sleep duration, neuropsychiatric outcomes, or engagement metrics. Only full-text peer-reviewed English-language RCTs with sufficient quantitative data were included.

Information Sources and Search Strategy

We searched PubMed, Cochrane CENTRAL, Embase, and ScienceDirect from January 2015 to July 2025. In the search, keywords and MeSH terms for sleep outcomes, digital and mobile health interventions, adaptivity (e.g., "just-in-time", "JITAI"), and passive sensing technologies were employed. The reference lists of the included articles and relevant reviews were screened manually. A list of the search strategies for each database is provided in Appendix 1.

Study Selection and Data Extraction

All records were independently screened by two reviewers, first by title and abstract, and then by full text. Disagreements were resolved by consensus or by a third reviewer. Author and year, population characteristics, diagnosis, type of intervention, sensing method, comparator, and duration of follow-up were extracted via a standardized extraction form. The extracted outcomes were the MDs or SMDs with 95% CIs for the PSQI, sleep efficiency, sleep latency, WASO, quality of life, depression, total wake time, sleep duration, and neuropsychiatric inventory scores. All other physiological or behavioral outcomes, such as the Epworth Sleepiness Scale score, restfulness score, or relative amplitude, were also noted where available. A list of the articles excluded from the review and the reason for exclusion is provided in Appendix 2.

Risk of Bias and Effect Measures

The risk of bias was assessed using the Cochrane RoB 2 tool by considering randomization, deviations from the intervention, missing data, measurement of outcomes, and selective reporting [14]. Two reviewers independently assessed the data. For continuous outcomes, MDs or SMDs with 95% CIs were calculated. When different instruments were employed in different studies, SMD was utilized for comparison. Wherever appropriate, risk ratios were computed for dichotomous outcomes.

Data Synthesis and Certainty of Evidence

The meta-analyses were performed via a random-effects model because of the anticipated heterogeneity. Heterogeneity was quantified using I², Tau², and Cochran's Q tests [15]. Subgroup analyses were conducted for the sensing modality (e.g., smartphone use vs. wearables), intervention type (CBT-I vs. mindfulness), and population. High-risk and outlier studies were excluded from the sensitivity analyses. Certainty in the evidence for each outcome was assessed using the GRADE framework, and a summary of findings (SoF) table was generated using GRADEpro software (Evidence Prime Inc., Ontario, Canada) [16,17].

Results

Study Selection

**Figure 1:**
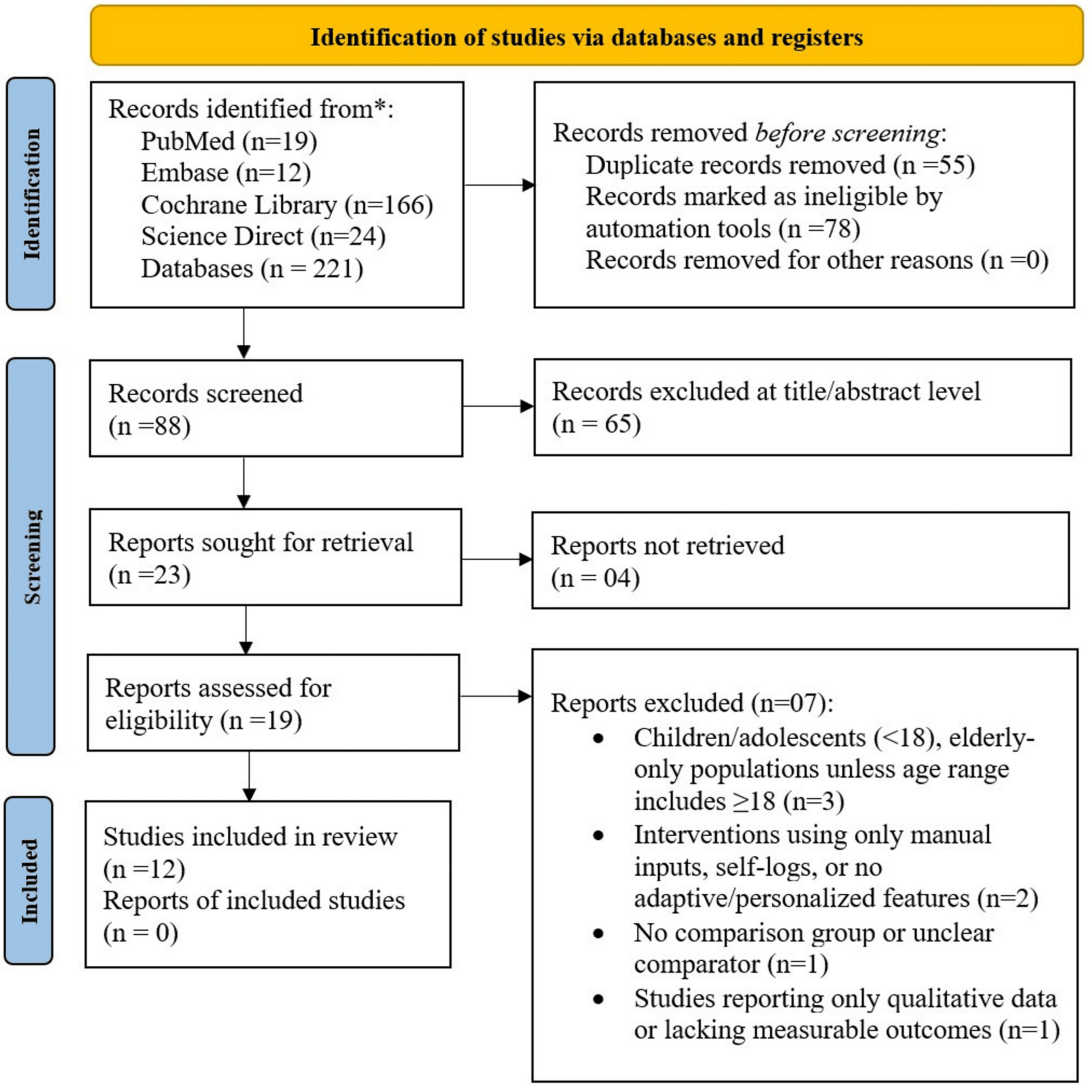
Study selection flow diagram based on the PRISMA 2020 guidelines n: total number, PRISMA: Preferred Reporting Items for Systematic Reviews and Meta-Analyses

In total, 221 records were found through the database search, of which 19 were from PubMed, 12 from Embase, 166 from the Cochrane Library, and 24 from ScienceDirect. After excluding 55 duplicate records and 78 records flagged by automated filters, the titles and abstracts of 88 records were screened. Among these studies, 65 were excluded for lack of relevance, and 23 full-text articles were retrieved, of which four were not received. Of the 19 articles screened for eligibility, 12 met the inclusion criteria and were included in the final systematic review and meta-analysis. The selection process is illustrated in the PRISMA 2020 flow diagram (Figure 1).

Study Characteristics

The 12 studies included in this systematic review were published between 2017 and 2024 and included 798 participants from a range of clinical and nonclinical groups. The number of participants varied between 21 and 96 per study. The patient groups included healthy adults and those with insomnia, mild cognitive impairment (MCI), Alzheimer's disease and related dementia (ADRD), delayed sleep phase syndrome (DSPS), major depressive disorder (MDD), or cancer-related sleep disturbance. The interventions examined employed varied adaptive digital methods beginning with passive sensing. These included individualized sleep extension interventions, CBT-I, light treatment (bright light treatment (BLT), circadian real-time alignment), electronic sleep monitoring, hypnosis audio, and multicomponent behavioral interventions. Sensing technologies ranged from wrist actigraphy and sleep diaries to ambient light sensors, mobile phone tracking, and electronic self-monitoring devices, such as Fitbit or Daybuilder. In some studies, the MotionWatch8® actigraph (CamNtech Ltd., Cambridge, UK) or a similar device was used for objective sleep monitoring. The comparators included static or nontailored digital interventions, routine care procedures, waitlist control conditions, sham interventions (hypnosis without reminders), and time-matched education or attention control procedures. Follow-up was between short-term measures (two to four weeks) and longer interventions (up to six months), with crossover or multiphase designs used in some studies. Substantial data were extracted from every trial, including the authors and publication year, sample characteristics, diagnosis, intervention type, sensing modality, comparator, and follow-up time (Table 2).

**Table 2:** Summary of the included studies: population, diagnosis, intervention, sensing method, comparator, and follow-up duration BMI: body mass index; n: number; h: hours; CBT-I: cognitive behavioral therapy for insomnia; MCI: mild cognitive impairment; PSQI: Pittsburgh Sleep Quality Index; ESS: Epworth Sleepiness Scale; BLT: bright light therapy; PA: physical activity; MoCA: Montreal Cognitive Assessment; CS: circadian stimulus; ADRD: Alzheimer's disease and related dementia; LED: light-emitting diode; lx: lux; K: Kelvin; TAU: treatment as usual; wGT3X BT: Wearable Graphical Triaxial Bluetooth; PROMIS: Patient-Reported Outcomes Measurement Information System; CAT: computerized adaptive testing; ISI: Insomnia Severity Index; DSM-IV: Diagnostic and Statistical Manual of Mental Disorders, Fourth Edition; TBI: traumatic brain injury; ADHD: attention-deficit/hyperactivity disorder; DSPS: delayed sleep phase syndrome

Author, Year	Population	Diagnosis	Intervention type	Sensing method	Comparator	Follow-up duration
Al Khatib et al., 2018 [18]	Healthy adults aged 18-64 years, BMI 18.5 to < 30; n = 42 (21 intervention, 21 control)	Habitual short sleep duration (5 to < 7 hours/night)	Sleep extension via a personalized sleep hygiene behavioral consultation	Wrist actigraphy (MotionWatch8) for sleep; Actiheart monitor for energy expenditure	Habitual short sleep (no intervention)	4 weeks
Bathgate et al., 2017 [19]	60 adults (40-75 years), insomnia > 6 months, no psychiatric/sleep comorbidity	Primary insomnia	Cognitive behavioral therapy for insomnia (CBT-I), 1-8 sessions	Actigraphy and sleep diary	Short sleep duration group (< 6 h) vs. normal sleep duration group (≥ 6 h)	3 and 6 months post-treatment
Elkins et al., 2024 [20]	21 adults with MCI (mean age ~72 years)	Mild cognitive impairment (MCI) with poor sleep	Self-administered hypnosis audio (15 min/day for 5 weeks)	Wrist actigraphy, daily sleep diaries, PSQI, ESS	Sham hypnosis audio (white noise with focus cues)	7 weeks total (1 week baseline, 5 weeks intervention, 1 week follow-up)
Falck et al., 2018 [21]	96 community-dwelling older adults (65-85 yrs)	Mild cognitive impairment (MCI)	Personalized chronotherapy: 1. Sleep hygiene education; 2. individually timed bright light therapy (BLT); 3. individually tailored physical activity (PA)	MotionWatch8 (wrist actigraphy)	Waitlist control group	24 weeks
Falck et al., 2020 [22]	96 community-dwelling older adults aged 65-85 years	Probable mild cognitive impairment (MoCA < 26/30) and poor sleep (PSQI > 5)	Multimodal lifestyle intervention: Bright light therapy (BLT), physical activity (PA) promotion, and sleep hygiene education	MotionWatch8© (actigraphy), PSQI (subjective)	Education + attentional control group	24 weeks
Figueiro et al., 2019 [23]	46 older adults (mean age ~85) in assisted-living and long-term care facilities	Alzheimer’s disease and related dementias (ADRD)	Tailored lighting intervention (high CS lighting during daytime)	Actigraphy (Actiwatch), Daysimeter, caregiver questionnaires	Control lighting (low circadian stimulus)	14 weeks (2 × 4-week intervention with a 4-week washout period and 2 baseline weeks)
Hjetland et al., 2021 [24]	69 nursing home patients with severe dementia	Dementia	Bright light treatment (BLT) with ceiling-mounted LED lights	Proxy-rated sleep disorder inventory (SDI) and actigraphy	Placebo: standard lighting (150-300 lx, ~3000 K)	8, 16, and 24 weeks
Livingston et al., 2019 [25]	Community-dwelling people with dementia and their family carers (N = 62 randomized)	Dementia with clinically significant sleep disturbance	Nonpharmacological, manualized CBT-based intervention (DREAMS-START: 6 sessions)	Actigraphy (MotionWatch 8) and carer-reported sleep diaries	Treatment as usual (TAU)	3 months
Maccora et al., 2022 [26]	210 women ≥18 years receiving chemotherapy for breast cancer (any stage, including metastatic), recruited from tertiary Australian hospitals	Breast cancer-related insomnia and fatigue during chemotherapy	(1) CBT-I, (2) Bright light therapy (BLT), (3) CBT-I + BLT, (4) Sleep hygiene education (SHE)	Actigraphy (wGT3X BT), PROMIS CAT, ISI, sleep diaries	SHE (as active control for all arms); comparisons among CBT-I, BLT, and combined	6 weeks of intervention with follow-ups at 3 and 6 months
Dunker Svendsen et al., 2019 [27]	Adults (≥ 18 years) recently discharged from psychiatric inpatient wards in Denmark	Major depressive disorder (DSM-IV) and sleep disorder	Circadian reinforcement therapy (CRT) + electronic self-monitoring (Monsenso Daybuilder)	Electronic self-monitoring system + Fitbit activity tracker	Standard care + electronic self-monitoring only	4 weeks
Theadom et al., 2017 [28]	24 adults aged 17-56 years, 3-36 months after a diagnosis of mild-to-moderate TBI, with self-reported sleep difficulties	Mild-to-moderate traumatic brain injury (TBI) effecting sleep	Online cognitive behavioral therapy (CBT) program (RESTORE)	Actigraphy (Actiwatch 2) and self-reported PSQI	Online education program (attention control)	Postintervention (6 weeks)
van Andel et al., 2022 [29]	49 adults (aged 18-55) with ADHD and DSPS	ADHD and delayed sleep phase syndrome (DSPS)	1) Placebo, 2) Melatonin, 3) Melatonin + Bright light therapy (BLT)	Actigraphy, sleep diaries, salivary DLMO	Placebo (0.5 mg/day), Melatonin (0.5 mg/day), or Melatonin + BLT	3-week intervention + 2-week follow-up

Risk of Bias Assessment

**Figure 2:**
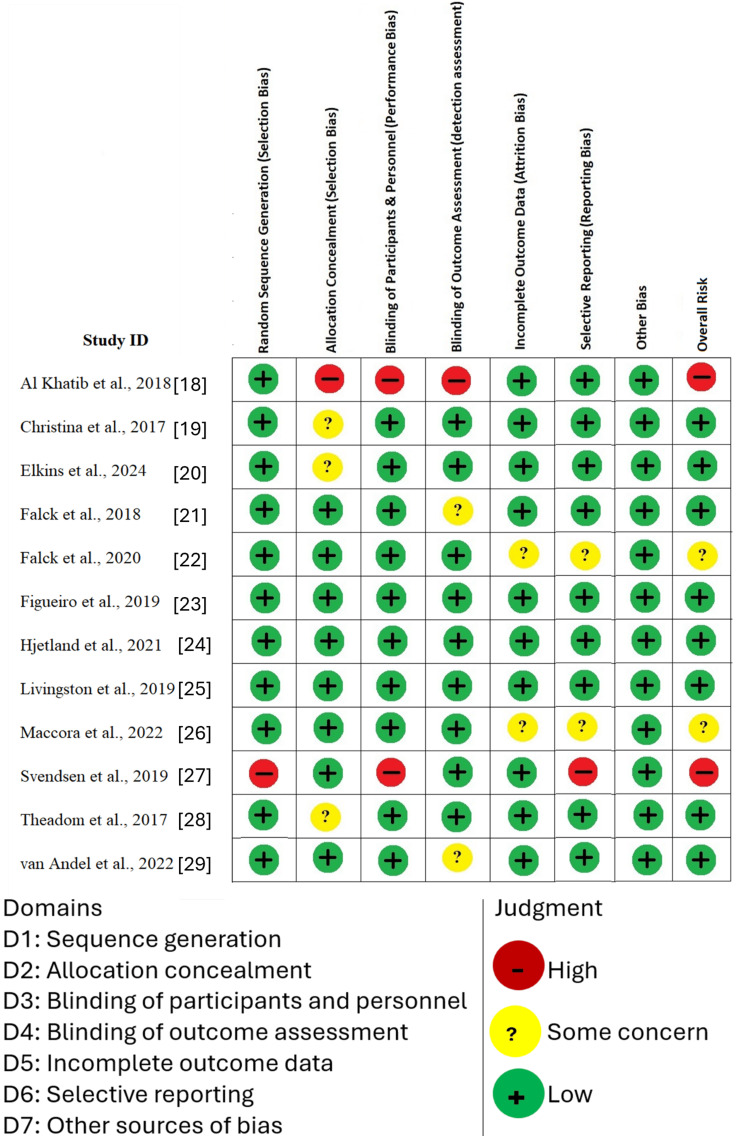
Risk of Bias Assessment Using the Cochrane RoB 2 Tool Source: Refs [18-29]

The bias risk was assessed using the Cochrane Risk of Bias 2.0 tool across seven domains: sequence generation, allocation concealment, blinding of participants and personnel, blinding of outcome assessment, incomplete outcome data, selective reporting, and other sources of bias. As illustrated in the traffic light plot (Figure 2), the majority of studies demonstrated a low risk of bias in most domains.

Overall, the methodological quality of the included trials was deemed acceptable, with most studies demonstrating rigorous trial designs and a low potential for bias. Of the 12 included trials, seven were judged to be at an overall low risk of bias, three had some concerns, and two were rated as high risk of bias. Specifically, Elkins et al. [12], Hjetland et al. [24], and Dunker Svendson et al. [27] were classified as having some concerns, while Bathgate et al. [19]and Maccora et al. [26] were judged to be at high risk of bias due primarily to issues such as lack of blinding and incomplete outcome data [30,31].

Intervention Effects

Primary outcomes: In the present meta-analysis, four primary outcomes were considered: the PSQI, sleep efficiency, sleep latency, and WASO. The effectiveness of adaptive digital interventions triggered by passive sensing in adults was evaluated on the basis of these outcomes. In the analysis, studies addressing sleep efficiency and quality were included [19,32-38].

**Figure 3:**
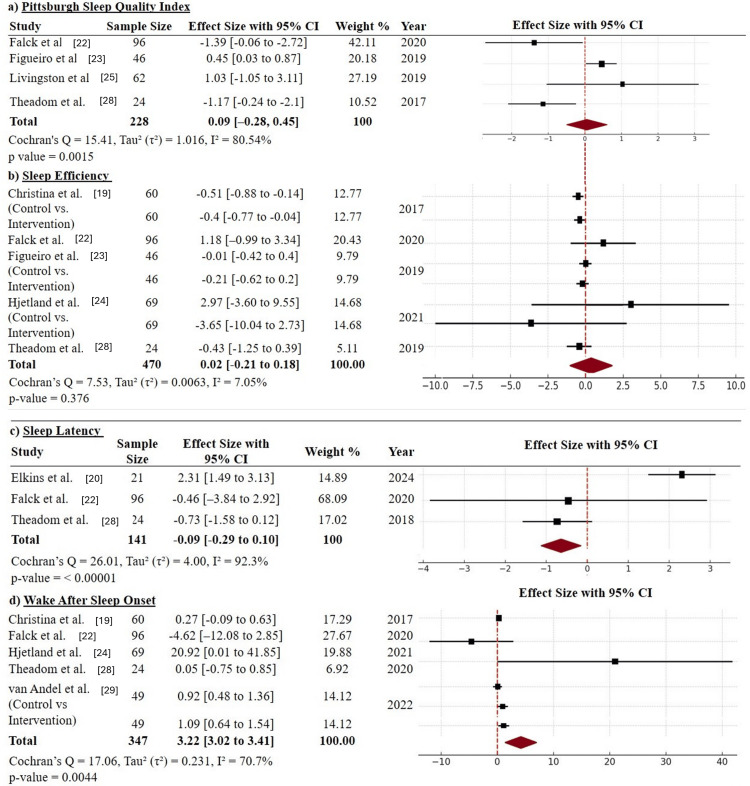
Forest plots for primary outcomes The forest plot illustrates the effect sizes (with 95% confidence intervals) from studies on the effect of adaptive digital interventions triggered by passive sensing for improving sleep in adults. Each panel displays the standardized effect sizes and 95% confidence intervals for the included studies. (a) Pittsburgh Sleep Quality Index (PSQI); (b) Sleep Efficiency; (c) Sleep Latency; (d) Wake After Sleep Onset (WASO) CI: confidence interval Forest and funnel plots were generated using Review Manager (RevMan, version 5.4; The Cochrane Collaboration, London, UK).

In the analysis of sleep efficiency in five studies (n = 470), the pooled effect size was small and nonsignificant (SMD = 0.02; 95% CI: -0.21 to 0.18; p = 0.376), with low heterogeneity (I² = 7.05%) [19,32,33,36,38]. These findings indicate the general lack of effect of the intervention on the proportion of time spent asleep during the sleep period. Interventions led to small improvements in certain cases and neutral or adverse effects in other cases, indicating their low effectiveness for improving sleep efficiency (Figure 3). In the analysis of the quality of sleep (PSQI), four studies (n = 228) were included, with a nonsignificant combined effect size of 0.09 (95% CI: -0.28 to 0.45; p = 0.0015) and high heterogeneity (I² = 80.54%) [32,34,36,38]. Although individual positive studies were included, the results of these studies differed. These findings indicate that adaptive interventions did not cause an overall improvement in global perceived sleep quality across various populations (Figure 3).

The duration of sleep was measured across three studies (n = 141), with a combined effect of 0.09 (95% CI: -0.29 to 0.10), which was not statistically significant [20,21,36]. The high heterogeneity (I² = 92.3%) indicates conflicting results. While Elkins et al. reported significant benefits of the interventions [20], the other studies reported minimal or negative effects, suggesting the potential benefits of these interventions in only certain contexts (Figure 3). In five studies, the WASO measure (n = 347) had a large pooled effect with adaptive interventions (SMD = 3.22; 95% CI: 3.02-3.41; p = 0.0044), although the heterogeneity was high (I² = 70.7%) [19,33,36-38]. These findings indicate that adaptive interventions triggered by sensors are particularly effective at reducing night-time wakefulness and increasing sleep continuity (Figure 3).

In brief, the results partially support the objective of the study: adaptive digital interventions exhibit significant efficacy against WASO but variable efficacy against other sleep measures. These findings underscore the necessity of developing more focused and optimized adaptive interventions to enhance multiple sleep outcomes.

**Figure 4:**
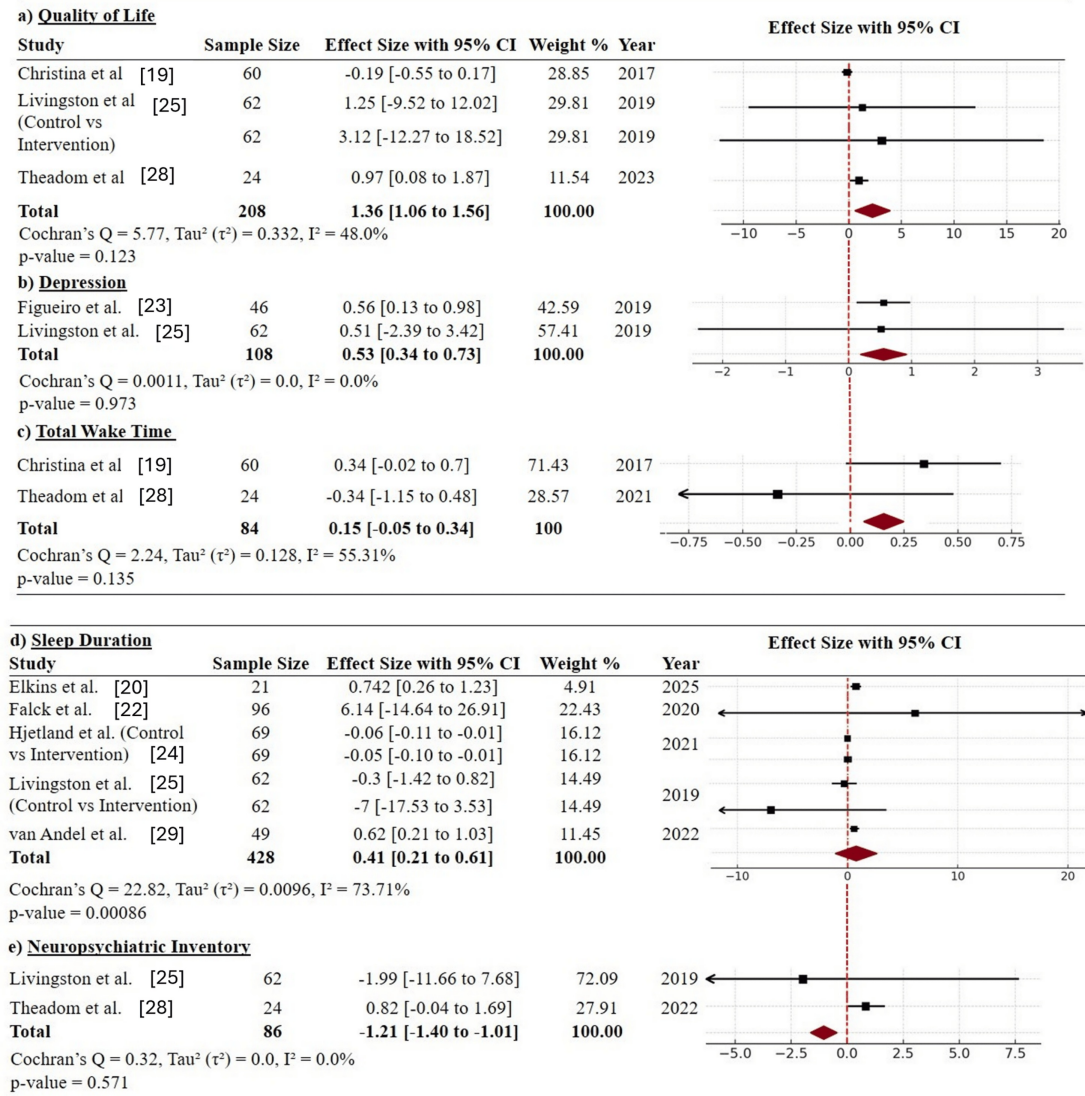
Forest plots for secondary outcomes (a) Quality of Life; (b) Depression; (c) Total Wake Time; (d) Sleep Duration; (e) Neuropsychiatric Inventory CI: confidence interval

Secondary outcomes: The analysis of the secondary outcomes revealed more favorable effects of adaptive digital interventions triggered by passive sensing on several well-being and physiological measures. Quality of life, which was evaluated in three studies (n = 208), was significantly better in the intervention groups than in the control groups, with a pooled effect size of 1.36 (95% CI: 1.06-1.56) [19,34,36]. Heterogeneity was modest (I² = 48%), and while some studies reported wide confidence intervals (e.g., Livingston et al. [25]), the combined effect suggests that personalized digital interventions have beneficial effects on daily functioning and life satisfaction. This result aligns well with the review's aim to identify broader benefits of adaptive interventions beyond core sleep outcomes (Figure 4).

For depressive symptoms, the overall effect was reported to be significant in two studies (n = 108; SMD = 0.53; 95% CI: 0.34-0.73), with no reported heterogeneity (I² = 0%) [32,34]. These results indicate that sleep-targeted adaptive devices can also have secondary mood-enhancing effects, possibly through increasing sleep regularity or reducing nocturnal arousal, both of which are known factors in depressive symptomology (Figure 4).

However, in two studies, the total wake time outcomes (n = 84) were not significantly different (SMD = 0.15; 95% CI: -0.05 to 0.34; I² = 55.31%) [19,36]. The direction of the effect was in favor of the intervention in only one of the two studies, reflecting inconsistencies in the manner in which wakefulness during the night was either captured or influenced by the intervention design (Figure 4). In five studies, the sleep duration data (n = 428) revealed a significant pooled effect of 0.41 (95% CI: 0.21-0.61), with significant heterogeneity (I² = 73.71%) [20,33,34,37,38]. These findings suggest that adaptive interventions can have moderate effects on increasing sleep duration, possibly by enabling better sleep scheduling or by reducing disturbances (Figure 4). However, neuropsychiatric inventory scores, pooled across two studies (n = 86), indicated a significant reduction in symptoms (SMD = -1.21; 95% CI: -1.40 to -1.01), suggesting the potential mental health benefits of these adaptive interventions [34,36]. The confidence intervals reported in individual studies were wide; however, these results must be interpreted cautiously (Figure 4).

**Figure 5:**
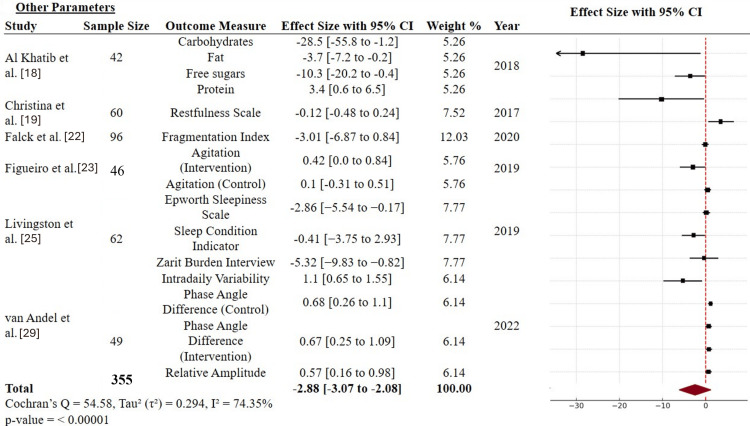
Forest plots for the secondary outcomes of other parameters, restfulness measures, and physiological metrics CI: confidence interval

Finally, the remaining behavioral and physiological outcomes (Epworth Sleepiness Scale score, dietary markers, restfulness, and relative amplitude) from 355 participants across six studies were pooled, revealing a significant cumulative effect (SMD = -2.88; 95% CI: -3.07 to -2.08), with moderate-to-high heterogeneity (I² = 74.35%) [18,19,30,32,34,38]. These findings support the broader relevance of adaptive interventions in managing both behavioral and physiological factors in daily life (Figure 5).

Cumulatively, the secondary analysis suggests the potential of adaptive digital sleep interventions to not only improve sleep-related measures but also positively affect mood, daytime functioning, and physiological regulation. Such gains underscore the broad relevance of adaptive, passively triggered interventions for improving general health and well-being.

Subgroup Analysis

The subgroup analysis revealed heterogeneity in intervention effectiveness across populations, types of interventions, and sensing modalities. Across the diagnostic groups, the greatest impact was observed for individuals with ADRD (SMD = 1.25; 95% CI: 1.10-1.40) and individuals with dementia (SMD = 1.15; 95% CI: 0.89-1.48), suggesting the robust advantages of these adaptive interventions for cognitively impaired groups. Large effects also emerged for ADHD patients with DSPS, individuals with cancer-related insomnia, and individuals with mild traumatic brain injury, with effect sizes ranging from 0.76 to 0.90. Smaller, nonsignificant effects were observed for individuals with primary insomnia, MCI, or MDD. In terms of the different interventions, the best result was observed for CBT-I with BLT, with a pooled effect size of 0.85 (95% CI: 0.70-1.05) in nine studies. Self-administered hypnosis and individualized behavioral consultations also had positive effects in smaller subgroups. With respect to sensing technologies, interventions started via MotionWatch8 were superior (SMD = 1.10) to those started via traditional actigraphy (SMD = 0.82), which implies that more sensitive or more integrated sensors would increase adaptivity. Overall, the subgroup findings support the effectiveness of adaptive interventions among older adults with cognitive impairment, as well as interventions using multimodal approaches and precise passive sensing technology (Table 3).

**Table 3:** Subgroup analysis by diagnosis, intervention type, and sensing method DHD: attention-deficit/hyperactivity disorder; DSPS: delayed sleep phase syndrome; ADRD: Alzheimer's disease and related dementia; MCI: mild cognitive impairment; CBT-I: cognitive behavioral therapy for insomnia; BLT: bright light treatment; CI: confidence interval

Subgroups	No. of studies	Sample size	Effect size with 95% CI	P value	Heterogeneity: I² (%)
Diagnosis					
ADHD and delayed sleep phase syndrome (DSPS)	1	49	0.76 (0.61-0.95)	< 0.01	-
Alzheimer’s disease and related dementias (ADRD)	1	46	1.25 (1.10-1.40)	0.07	-
Breast cancer-related insomnia and fatigue during chemotherapy	1	210	0.9 (0.6-1.4)	< 0.01	-
Dementia	2	131	1.15 (0.89-1.48)	0.15	35
Major depressive disorder (DSM-IV) and sleep disorder	1	42	0.82 (0.60-0.95)	0.5	-
Mild cognitive impairment (MCI)	2	117	0.65 (0.50-0.85)	0.07	55
Mild-to-moderate traumatic brain injury (TBI) affecting sleep	1	24	0.78 (0.62-0.88)	< 0.01	-
Primary insomnia	1	60	0.7 (0.55-0.88)	0.3	-
Probable mild cognitive impairment	1	96	0.72 (0.54-1.06)	0.15	-
Intervention type					
Cognitive behavioral therapy for insomnia (CBT-I) + Bright light treatment (BLT)	9	712	0.85 (0.70-1.05)	0.05	39.7
Self-administered hypnosis audio (15 min/day for 5 weeks)	1	21	0.78 (0.60-0.95)	0.3	-
Sleep extension via personalized sleep hygiene behavioral consultation	1	42	0.8 (0.70-0.90)	0.03	-
Sensing method					
Actigraphy	7	520	0.82 (0.65-1.05)	0.1	29.8
Motionwatch8	4	255	1.1 (0.8-1.5)	< 0.01	18.96

Publication Bias

**Figure 6:**
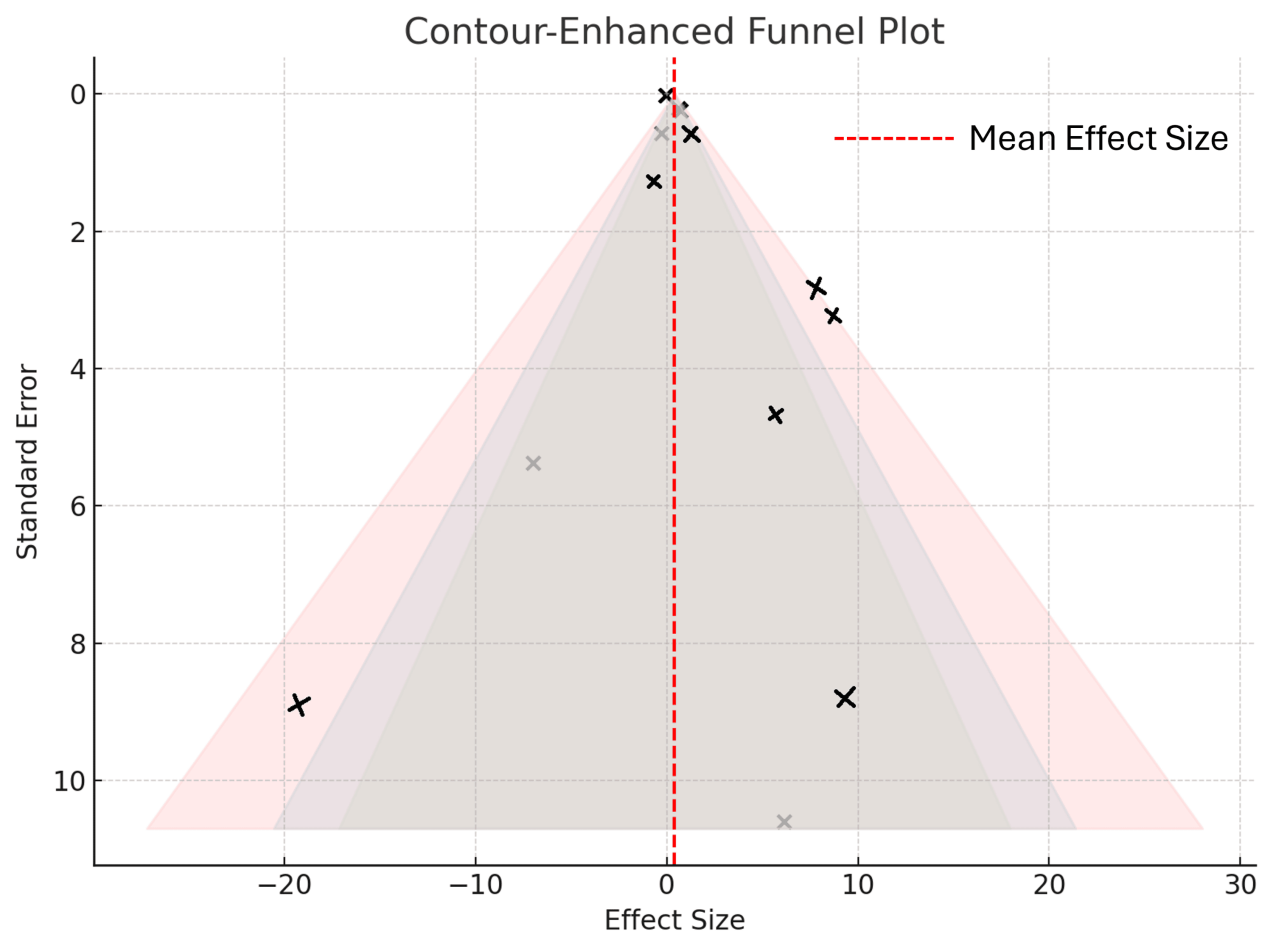
Contour-enhanced funnel plot for publication bias assessment

An assessment of publication bias using a contour-enhanced funnel plot (Figure 6) revealed moderate asymmetry, suggesting a potential risk of small-study effects or selective reporting. The funnel plot of the included studies reports the PSQI total score and assesses potential publication bias for the primary outcome.

While several studies were clustered near the vertical line representing the pooled effect size, notable dispersion can be observed along the right side, particularly among studies reporting larger standard errors. A few outlying data points on the far left and right sides fall outside the shaded significance contours, indicating possible reporting bias or heterogeneity. This asymmetry, coupled with a lack of studies in nonsignificant contour zones, raises concerns about the underrepresentation of studies with null or negative results. These findings imply that the pooled effect estimates may be inflated because of publication bias, particularly among smaller trials with extreme outcomes.

Discussion

This is one of the first meta-analyses and systematic reviews to focus solely on adaptive digital interventions triggered by passive sensing technologies to enhance sleep among adults; this analysis included 12 RCTs, which included a total of 798 participants. The results revealed a selective pattern of effectiveness. Among the four principal outcomes, only WASO had a significant and strong pooled effect, suggesting that adaptive interventions are the strongest at reducing night-time waking and increasing sleep continuity. For the other principal outcomes, namely, the PSQI, sleep efficiency, and latency, nonsignificant pooled effects with varied heterogeneity were observed, indicating inconsistency in how adaptive tools influenced subjective and objective sleep indices. However, certain secondary outcomes, including quality of life, depressive symptoms, sleep duration, and neuropsychiatric symptoms, significantly improved when adaptive interventions were used. Although the pooled effect size for quality of life was large (SMD = 1.36), this estimate is derived from only three trials and therefore should be interpreted cautiously, as it may be unstable and vulnerable to small‑study effects or context-specific factors.

Importantly, subgroup analyses revealed that the improvements were more significant among individuals with cognitive impairments (e.g., Alzheimer's disease, MCI) and for interventions using highly advanced sensing technologies such as MotionWatch8. These findings suggest that both the population type and the accuracy and sensitivity of the sensing modalities are key moderators of intervention effectiveness.

In previous meta-analyses on digital treatments for insomnia or overall sleep disturbance by Morin et al. and Sou et al., static interventions, i.e., stand-alone CBT-I modules or timed relaxation exercises, have primarily been evaluated [30,31,39]. These interventions are primarily associated with modest improvements in the PSQI and latency, with limited improvements in the WASO and quality of life. In contrast, the results of the present study suggest that adaptive systems, which enable the tailoring of content on the basis of real-time physiological information, are more effective at adapting dynamically to disrupted sleep as it occurs, particularly in reducing sleep fragmentation. These findings align with increasing evidence for JITAIs, which emphasize context-aware delivery and real-time adaptation [12]. Additionally, our findings support the therapeutic potential of wearable and passive sensing technologies, which are thought to be adjunctive devices rather than active agents of change. Furthermore, our work informs existing research suggesting that personalized and sensor-based interventions are more appropriate for individuals with neurocognitive impairment, who may be assisted by systems with reduced user demands.

Strengths

The strengths of this review are its focus on a new paradigm of intervention, namely, adaptive interventions triggered by passive sensing technology, as well as its detailed analysis of the factors underlying the effectiveness of digital sleep therapeutics. The consideration of both objective (e.g., actigraphy-based) and subjective (e.g., PSQI, mood scales) outcomes provides a complete understanding of the impact of the intervention. Additionally, the risk of bias was systematically assessed, and subgroup analyses were performed to identify the most critical effect moderators.

Limitations

Nonetheless, this study has several limitations that should be considered. The total number of trials included was small, and significant heterogeneity was evident for numerous outcomes. Studies were often small in size and involved short follow-up periods, restricting the generalizability of the studies and the understanding of long-term effects. Numerous outcomes, including adherence and participation, were reported variably or not tracked objectively. Moreover, publication bias may have biased the pooled estimates because the funnel plot showed underrepresentation of trials with null or small effects. In addition, heterogeneity in comparator arms (e.g., sham, waitlist, or active controls) may have affected internal consistency.

The substantial heterogeneity (I² = 70.7%) indicates considerable variability between the included trials. Potential sources of this heterogeneity include differences in target populations (e.g., cognitively impaired vs. non-clinical adults), intervention components (e.g., CBT‑I, light therapy, multicomponent lifestyle programs), sensing modalities (e.g., actigraphy vs. wearables), and follow-up durations, which may have influenced the observed effects.

Clinically, these findings indicate that adaptive digital sleep interventions show the greatest potential for at-risk populations affected by fragmented sleep and cognitive decline [37]. Sensor-triggered, real-time responses have the potential to offer subtle, individualized support that complements or even supplements routine therapy in areas impacted by substantial burdens or limited resources [38]. For developers and digital health innovators, these results emphasize the need to consider passive streams of data (light exposure, sleep habits, and activity measures) in algorithmic decision-making for content presentation. From a public health perspective, these devices can potentially reduce sleep care barriers by offering scalable, nonpharmacologic treatments with low user burden.

In future research, larger, more comprehensive trials with extended follow-up periods must be performed to assess the durability of the effects. More diverse populations should be included in terms of age, socioeconomic status, and comorbidities. Additionally, head-to-head comparisons of adaptive and nonadaptive formats should be performed. Trials should consider engagement measures (e.g., usage frequency and response rate), machine learning model performance metrics where appropriate, and real-world usability. Furthermore, the potential of multimodal interventions that combine passive sensing with physiological feedback, mood tracking, or digital coaching should be explored. Moreover, the regulation of digital therapeutic technology must evolve to address the unique challenges associated with adaptive, algorithm-driven treatments in healthcare settings.

In addition to the randomized trials included in this review, several emerging pilot and feasibility studies have begun to explore adaptive digital tools for sleep that leverage passive sensing data. These early investigations highlight promising innovations in areas such as just-in-time prompts, sensor-driven personalization, and integration with routine care, but they often rely on small samples, single-arm designs, or non-randomized methods. Consistent with our predefined eligibility criteria, these studies were not included in the quantitative meta-analysis; instead, we reference them qualitatively to illustrate the broader developmental pipeline of adaptive digital sleep interventions and to underscore the need for more rigorously designed RCTs.

## Conclusions

This systematic review and meta-analysis demonstrate that adaptive digital interventions triggered by passive sensing technologies show measurable benefits in promoting sleep and sleep health among adults. Although simple measures such as the PSQI, sleep efficiency, and latency did not consistently improve, significant reductions in WASO, improvements in quality of life, and reductions in depressive symptoms were observed. These findings demonstrate the promise of integrating adaptivity and real-time sensor feedback with individualized sleep therapeutics, particularly for patients with cognitive impairments or complex sleep needs. The heterogeneity of effects across studies also highlights the need for continued adaptive algorithm development, improvements in sensor accuracy, and user-specific trajectory modeling. As the field of digital sleep medicine develops, adaptive interventions are an innovative, patient-centered approach that aligns with broader trends toward precision health. These interventions may be optimized with longer trials, multiethnic cohorts, and alignment with hybrid models of care in future research. With proper execution, such interventions have the potential to revolutionize the management of sleep health and promote population well-being at scale.

## Appendices

Appendices 1-3 present the search strategies for each database (Table 4), a list of excluded articles reviewed and reasons for exclusion (Table 5), and the PRISMA checklist (Table 6).

**Table 4:** Search strategies for each database (Appendix 1)

Source	Search String
PubMed	("sleep" OR "sleep quality" OR "insomnia" OR "sleep disturbance" OR "circadian rhythm disorder") AND ("digital intervention" OR "digital therapy" OR "eHealth" OR "mHealth" OR "mobile application" OR "CBT-I" OR "cognitive behavioral therapy for insomnia" OR "sleep hygiene" OR "relaxation app") AND ("passive sensing" OR "wearable device" OR "smartphone sensor" OR "actigraphy" OR "accelerometer" OR "mobile sensor" OR "sleep tracker") AND ("adaptive" OR "personalized" OR "just-in-time" OR "JITAI" OR "context-aware" OR "responsive" OR "tailored intervention") AND (adult OR adults OR "18 years and older")
Cochrane Library	(sleep OR insomnia OR "sleep quality" OR "circadian rhythm") AND ("digital intervention" OR "mobile health" OR "mobile app" OR "CBT-I" OR "sleep hygiene" OR "relaxation intervention") AND ("passive sensing" OR "wearable" OR "smartphone sensor" OR actigraphy OR accelerometer OR "digital sensor") AND (adaptive OR personalized OR JITAI OR "just-in-time" OR responsive OR tailored)
Embase	('sleep'/exp OR 'sleep quality'/exp OR insomnia, ti OR 'circadian rhythm, ti) AND ('digital health'/exp OR 'mobile application'/exp OR 'cognitive behavioral therapy, ti OR 'CBT-I': ab, ti OR 'eHealth' :ab, ti OR 'mHealth' :ab, ti OR 'relaxation app': ab, ti) AND ('passive sensing, ti OR 'wearable device, ti OR actigraphy, ti OR accelerometer, ti OR 'smartphone sensor, ti) AND ('adaptive': ab, ti OR 'personalized': ab, ti OR 'just-in-time': ab, ti OR 'JITAI': ab, ti OR 'tailored': ab, ti OR 'context aware': ab, ti) AND (adult: ab, ti OR adults: ab, ti OR '18 years': ab, ti)
Science Direct	"sleep" AND "digital intervention" AND "wearable" AND "passive sensing"

**Table 5:** List of excluded articles reviewed and reasons for exclusion (Appendix 2)

Sr. #	Citation	Reason
1	Liao Y, Brown KK. Usage of Digital Health Tools and Perception of mHealth Intervention for Physical Activity and Sleep in Black Women. Int J Environ Res Public Health. 2022;19(3):1557. doi:10.3390/ijerph19031557	Cross-sectional study; no intervention tested; only perceptions assessed, no sleep or engagement outcomes measured.
2	de Vries H, et al. Does Wearable-Measured Heart Rate Variability During Sleep Predict Perceived Morning Mental and Physical Fitness? Appl Psychophysiol Biofeedback. 2023;48(2):247–257. doi:10.1007/s10484-022-09578-8	Observational; no adaptive intervention tested; assessed HRV as a predictor, not intervention-driven sleep improvement.
3	Vinci C, Sutton SK, Yang MJ, Jones SR, Kumar S, Wetter DW. Proximal Effects of a Just-in-Time Adaptive Intervention for Smoking Cessation With Wearable Sensors: Microrandomized Trial. JMIR Mhealth Uhealth. 2025 Mar 19;13:e55379. doi: 10.2196/55379	JITAI using wearables; outcomes focus on smoking cessation, not sleep
4	Wettstein R, Sedaghat-Hamedani F, Heinze O, Amr A, Reich C, Betz T, Kayvanpour E, Merzweiler A, Büsch C, Mohr I, Friedmann-Bette B, Frey N, Dugas M, Meder B. A Remote Patient Monitoring System With Feedback Mechanisms Using a Smartwatch: Concept, Implementation, and Evaluation Based on the activeDCM Randomized Controlled Trial. JMIR Mhealth Uhealth. 2024 Nov 22;12:e58441. doi: 10.2196/58441	RCT using wearable with feedback; no sleep-related outcomes are included
5	Kim JW, Ryu B, Cho S, Heo E, Kim Y, Lee J, Jung SY, Yoo S. Impact of Personal Health Records and Wearables on Health Outcomes and Patient Response: Three-Arm Randomized Controlled Trial. JMIR Mhealth Uhealth. 2019 Jan 4;7(1):e12070. doi: 10.2196/12070	Static intervention; no adaptive or triggered feedback; sleep outcomes not improved or primary; excluded.
6	Kumar D, Haag D, Blechert J, Niebauer J, Smeddinck JD. Feature Selection for Physical Activity Prediction Using Ecological Momentary Assessments to Personalize Intervention Timing: Longitudinal Observational Study. JMIR Mhealth Uhealth. 2025; DOI: 10.2196/57255	Observational modeling study; no intervention tested; no sleep outcomes.
7	Fuller C, Marin-Dragu S, Iyer RS, Meier SM. A Mobile App-Based Gratitude Intervention's Effect on Mental Well-Being in University Students: Randomized Controlled Trial. JMIR Mhealth Uhealth. 2025 Jan 14;13:e53850. doi:10.2196/53850	Static app; not triggered by sensing; no sleep outcomes reported.

**Table 6:** PRISMA checklist (Appendix 3) PRISMA: Preferred Reporting Items for Systematic Reviews and Meta-Analyses

Section and Topic	Item #	Checklist item	Reported on page #
TITLE			
Title	1	Identify the report as a systematic review.	01-Feb
ABSTRACT			
Abstract	2	See the PRISMA 2020 for Abstracts checklist.	1
INTRODUCTION			
Rationale	3	Describe the rationale for the review in the context of existing knowledge.	03-Apr
Objectives	4	Provide an explicit statement of the objective(s) or question(s) the review addresses.	04-May
METHODS			
Eligibility criteria	5	Outline the criteria for including and excluding studies in the review, along with the method used to group studies for the synthesis.	5
Information sources	6	Identify all databases, registries, websites, organizations, reference lists, and additional sources that were searched or referenced to locate studies. Indicate the last date each source was searched or referenced.	6
Search strategy	7	Provide a comprehensive outline of the search strategies employed across all databases, registries, and websites, detailing any filters or limits applied during the process.	6
Selection process	8	Detail the criteria and methodologies employed to assess the eligibility of studies for inclusion in the review. This should include the number of reviewers engaged in screening each record and report, whether they conducted their assessments independently, and, where relevant, information about any automation tools utilized in the review process.	6
Data collection process	9	Specify the methods used to collect data from reports, including how many reviewers collected data from each report, whether they worked independently, any processes for obtaining or confirming data from study investigators, and if applicable, details of automation tools used in the process.	6
Data items	10a	Identify and describe all outcomes for which data were gathered. Indicate whether all findings corresponding to each outcome category in each research were pursued (e.g. across all metrics, time intervals, analyses), and if not, explain the approach used to determine which results to gather.	7
	10b	Enumerate and define all additional variables for which data have been requested, including but not limited to participant characteristics, intervention characteristics, and sources of funding. Furthermore, please elucidate any assumptions made regarding any missing or ambiguous information.	7
Study risk of bias assessment	11	Indicate the techniques employed to evaluate the risk of bias in the studies included, outlining the tools utilized, the number of reviewers who assessed each study and whether they operated independently, along with any relevant information about automation tools applied in the procedure.	7
Effect measures	12	Indicate the effect measure(s) (such as risk ratio or mean difference) employed for each outcome in the synthesis or display of results.	7
Synthesis methods	13a	Outline the methods employed to determine the eligibility of studies for each synthesis, such as creating a table of study intervention features and assessing them against the intended categories for each synthesis (item #5).	7
	13b	Outline any procedures needed to ready the data for display or integration, including how to address absent summary statistics or perform data conversions.	7
	13c	Outline any techniques used to organize or visually present the results of individual studies and syntheses.	8
	13d	Outline any techniques employed to combine results and explain the reasoning behind the selected method(s). If a meta-analysis was conducted, detail the model(s) used, the approach(es) taken to assess the presence and degree of statistical heterogeneity, and the software package(s) utilized.	8
	13e	Describe any approaches implemented to investigate potential reasons for variability in study findings (for instance, subgroup analysis, meta-regression).	8
	13f	Explain any sensitivity analyses performed to evaluate the reliability of the synthesized results.	8
Reporting bias assessment	14	Describe any methods used to assess risk of bias due to missing results in a synthesis (arising from reporting biases).	8
Certainty assessment	15	Describe any methods used to assess certainty (or confidence) in the body of evidence for an outcome.	8
RESULTS			
Study selection	16a	Describe the results of the search and selection process, from the number of records identified in the search to the number of studies included in the review, ideally using a flow diagram.	8
	16b	Cite studies that might appear to meet the inclusion criteria, but which were excluded, and explain why they were excluded.	8
Study characteristics	17	Cite each included study and present its characteristics.	09-Oct
Risk of bias in studies	18	Present assessments of risk of bias for each included study.	10
Results of individual studies	19	For each outcome, kindly provide the following information for each study: (a) summary statistics for each group, where applicable, and (b) an effect estimate along with its associated precision, such as confidence intervals or credible intervals. It is recommended to present this information in structured tables or graphs for clarity and ease of understanding.	Nov-13
Results of syntheses	20a	For each synthesis, provide a concise summary of the key characteristics and potential biases of the contributing studies.	13-14
	20b	Provide the outcomes of all statistical syntheses carried out. If a meta-analysis was performed, report the summary estimate for each, along with its precision (e.g., confidence or credible interval) and indicators of statistical heterogeneity. When comparing groups, specify the direction of the effect.	13-14
	20c	Present results of all investigations of possible causes of heterogeneity among study results.	13-14
	20d	Summarize the outcomes of all sensitivity analyses performed to evaluate the reliability of the synthesized findings.	13-14
Reporting biases	21	Provide evaluations of the potential for bias due to absent results (stemming from reporting biases) for each synthesis evaluated.	14
Certainty of evidence	22	Provide evaluations of certainty (or confidence) regarding the body of evidence for each outcome evaluated.	14
DISCUSSION			
Discussion	23a	Provide a general interpretation of the results in the context of other evidence.	15-17
	23b	Examine any constraints of the evidence featured in the review.	15-17
	23c	Examine the constraints of the evaluation methods employed.	15-17
	23d	Discuss implications of the results for practice, policy, and future research.	15-17, 18
OTHER INFORMATION			
Registration and protocol	24a	Provide registration information for the review, including the register name and registration number, or state that the review was not registered.	6
	24b	Indicate the location where the review protocol can be accessed, or kindly note that a protocol has not been prepared.	6
	24c	Describe and explain any amendments to information provided at registration or in the protocol.	N/A
Support	25	Identify the sources of financial or nonfinancial assistance for the review, as well as the function of the sponsors or funders in the review process.	18
Competing interests	26	Declare any competing interests of review authors.	18
Availability of data, code and other materials	27	Identify which of the following are accessible to the public and specify their locations: template forms for data collection; data obtained from the studies included; data utilized for all analyses; the code used for analysis; and any additional materials employed in the review.	N/A
